# Synergistic Effects of Salvianolic Acid B and Puerarin on Cerebral Ischemia Reperfusion Injury

**DOI:** 10.3390/molecules23030564

**Published:** 2018-03-02

**Authors:** Chengli Ling, Jianming Liang, Chun Zhang, Ruixiang Li, Qianqian Mou, Jin Qin, Xiaofang Li, Jianxin Wang

**Affiliations:** 1 School of Pharmacy, Chengdu University of Traditional Chinese Medicine, Chengdu 610072, China; l289632020@sina.com (C.L.); qqmou22@163.com (Q.M.); 2 Department of Pharmaceutics, School of Pharmacy, Fudan University & Key Laboratory of Smart Drug Delivery, Ministry of Education, Shanghai 201203, China; 14111030024@fudan.edu.cn (J.L.); zhangchun8704@163.com (C.Z.); pharmlrx@sina.com (R.L.); qinjingyx@sina.com (J.Q.)

**Keywords:** Salvianolic acid B, puerarin, ischemia stroke, combination, synergistic effect, inflammation

## Abstract

Ischemic stroke (IS) is characterized by the sudden loss of blood circulation to an area of the brain, resulting in a corresponding loss of neurologic function. It has been a worldwide critical disease threatening to the health and life of human beings. Despite significant progresses achieved, effective treatment still remains a formidable challenge due to the complexity of the disease. Salvianolic acid B (Sal-B) and Puerarin (Pue) are two active neuroprotectants isolated from traditional Chinese herbs, Salvia miltiorrhiza and Kudzu root respectively, which have been used for the prevention and treatment of IS for thousands of years in China. The activities of two compounds against cerebral ischemia reperfusion injury have been confirmed via various pathways. However, the therapeutic efficacy of any of the two components is still unsatisfied. In the present study, the effect of the combination of Sal-B and Pue on IS was evaluated and validated in vitro and in vivo. The ratio of two compounds was firstly optimized based on the results of CoCl_2_ damaged PC12 cells model. The co-administration exhibited significantly protective effect in CoCl_2_ induced PC12 cells injury model by reducing ROS, inhibiting apoptosis and improving mitochondrial membrane potential in vitro. Moreover, Sal-B + Pue significantly relieved neurological deficit scores and infarct area than Sal-B or Pue alone in vivo. The results indicated that neuroprotection mechanism of Sal-B + Pue was related to TLR4/MyD88 and SIRT1 activation signaling pathway to achieve synergistic effect, due to the inhibition of NF-κB transcriptional activity and expression of pro-inflammatory cytokine (TNF-α, IL-1β, IL-6). In conclusion, the combination of Sal-B and Pue exerted much stronger neuroprotective effect than Sal-B or Pue alone, which provides a potential new drug and has great significance for the treatment of IS.

## 1. Introduction

Cerebral ischemia reperfusion, namely ischemia stroke (IS), is a common refractory disease with a serious hazard to human health and a leading cause of death worldwide [1]. IS involves death of brain tissue (cerebral infarction) resulting from inadequate supply of blood and oxygen due to the blockage of brain blood vessels. A series of pathophysiologic events, such as energy metabolism disorder, glutamate excitotoxicity, ion balance disorder, oxidative stress, inflammation and apoptosis, will occur because of the blocked blood supply [2]. Currently, thrombolytic agents have been used for clinical treatment of IS by conversing inactive plasminogen to plasmin and improving cerebral blood flow (CBF). However, the efficacy is limited owing to the narrow therapeutic time window and various safety problems [3]. So, there is an urgent need for the development of novel effective drugs for the treatment of IS.

Traditional Chinese Medicine (TCM) has been applied for thousands of years and accumulated extensive clinical experience in treating IS with indigenous herbal medicines [4]. Salvia miltiorrhiza and Kudzu root, in Chinese, Danshen and Gegen, are two mostly used herbal drugs to prevent and treat IS in TCM and have been often using as a pair. They composed the DanGe formula and are the main components of Tongmai formula [5], which are classical clinical prescriptions and have a long history for the treatment of IS. According to the theory of TCM, the two herbs can work on multi-targets and different pathways through synergistic mechanism [6]. The neuroprotective effect of Gegen plus danshen intravenous injections on vascular endothelial functioning has been confirmed for IS [7].

**Figure 1:**
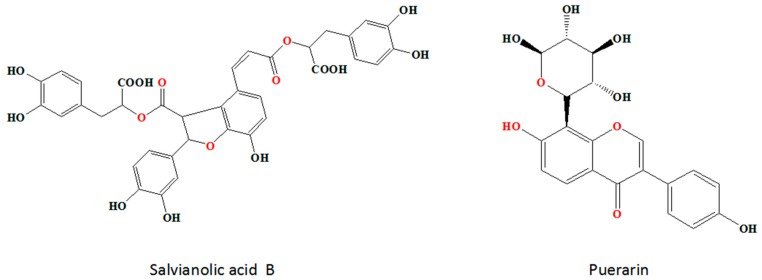
The chemical structures of Sal-B and Pue.

Salvianolic acid B (Sal-B) and puerarin (Pue) (in Figure 1) are the main active components of Salvia miltiorrhiza and Kudzu root, respectively [8,9]. It has been proved that both of them have significant therapeutic effect against IS. Pue protects against ischemic brain injury in a rat model of transient focal ischemia [10] and confers a neuroprotection by attenuating autophagy in neurons but not in astrocytes [11]. Sal-B has been proved having anti-inflammatory and neuroprotective effects against IS insults in vitro and in vivo, which is associated with the inhibition of TLR4 signaling [12]. Sal-B exerts neuroprotective effect against IS in rats via the NF-κB pathway associated with suppression of platelets activation and neuroinflammation [13] and also increases the level of antioxidant substances and decreases the production of free radicals, particularly hydroxyl radicals [14].

However, the bioavailability of both Sal-B and Pue via oral administration is very poor. They are difficult to absorb into the blood to exert protective effect because of the low stability in GI tract and low permeability of Sal-B and poor solubility of Pue. Thus, the injection of Sal-B or Pue have been developed and used in the clinical treatment of IS in China for more than 30 years with good efficacy and low side-effects [15]. The clinical application results were presented to demonstrate co-administration of Pue injection and Danshen injection have obvious improvement of neurologic injury in 36 patients with acute cerebral infarction [16] and research indicates that Danshen injection and Pue injection have a better anti-inflammatory than Sal-B or Pue and can further enhance treatment outcome [17]. However, to our knowledge, the mechanisms of the compatibility of Sal-B and Pue are not clear and the optimal ratio of the two active compounds has not been studied yet. This greatly restricts the co-administration of them and hinders to realize better therapeutic effect in clinic.

IS is commonly accompanied by neuroinflammatory response, resulting in over-release of inflammatory chemokines, cytokines and adhesion molecules, which would further deteriorate brain injury [18]. Toll-like receptor 4 (TLR4) is a membrane protein receptor expressed in neuron cells and responsible for inflammatory response [19]. The over-expressed TLR4 often activates the TLR4 adaptor protein myeloid differentiation primary response gene 88 (MyD88) [20]. The TLR4/MyD88 signaling will further stimulate a complex including interleukin-1 receptor-associated kinase 1(IRAK1) and TNF receptor-associated factor 6 (TRAF6) [21]. Finally, this signal increases the level of nuclear factor kappa B (NF-κB) transcriptional activity and inflammatory factors in ischemic brain. Silent information regulator 1 (SIRT1) is a histone deacetylase, whose activity is dependent on nicotinamide adenine dinucleotide (NAD+). SIRT1 can inhibit p53 and NF-κB induce inflammatory pathway by deacetylation [22]. The signal pathway of TLR4/MyD88 and SIRT1 activation in penumbra is a main cause of neuroinflammation of IS [23,24], which represents potentially neuroprotective therapeutic targets for the treatment of the disease. Recent study showed that Pue protects brain tissue against I/R injury by inhibiting the inflammatory response of TLR4/MyD88 signaling pathway [25]. Sal-B can protect against acute ethanol-induced liver injury via SIRT1 activation [26] and attenuate apoptosis and inflammation via activating SIRT1 in experimental stroke rats [27], indicating that the neuroprotection of Sal-B is related to the inhibition of SIRT1/NF-κB pathway. Therefore, the two compounds may affect through different way and thus lead to better outcome when used together.

In the present study, we intended to explore the optimum ratio of Sal-B and Pue, verify the synergistic effect of them on the inhibition of neuroinflammation and ameliorating brain injury after ischemia stroke. Furthermore, the pharmacological mechanisms of the combination of Sal-B and Pue were elucidated through TLR4/MyD88 and SIRT1 activation signaling pathway.

## 2. Results

### 2.1. Sal-B, Pue and Sal-B + Pue Increased Cells Viabilities on Cobalt Chloride (CoCl_2_) Induced PC12 Cells Injury

**Figure 2:**
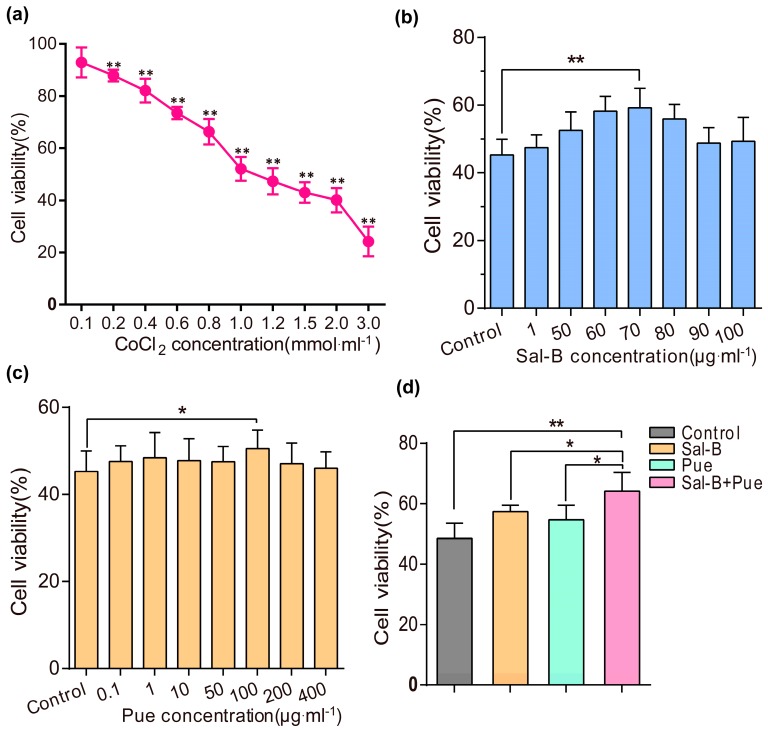
Protective effects of Sal-B, Pue and Sal-B + Pue in CoCl_2_ injured PC12 cells. (**a**) The viability of PC12 cells induced with different concentration (0.1, 0.2, 0.4, 0.6, 0.8, 1, 1.2, 1.5, 2, 3 mmol·L^−1^) of CoCl_2_ for 24 h (** *p* < 0.01 vs. control group); (**b**) The viability of PC12 cells pretreated with various concentrations of Sal-B (1, 50, 60, 70, 80, 90, 100 μg·mL^−1^) and (**c**) Pue (0.1, 1, 10, 50, 100, 200, 400 μg·mL^−1^) for 1 h before induced by CoCl_2_ (* *p* < 0.05; ** *p* < 0.01 vs. control); (**d**) The viability of PC12 cells pretreated with Sal-B (70 μg·mL^−1^) and Pue (100 μg·mL^−1^) simultaneously for 1 h before induced by CoCl_2_. Data were expressed as mean ± SD (*n* = 6), * *p* < 0.05 vs. Sal-B or Pue; ** *p* < 0.01 Sal-B + Pue vs. blank control.

The CoCl_2_ damaged PC12 cells model was firstly established. As shown in Figure 2a, the PC12 cells viability presented in a concentration-dependent manner when induced by CoCl_2_ at different concentration for 24 h. It could be find that the viability of PC12 cells was lower than 50% (47.3 ± 5.04%) when the concentration of CoCl_2_ was set as 1.2 mmol·L^−1^. Thus, the concentration was chosen for the following studies.

To investigate the optimal ratio of Sal-B and Pue, the cytotoxicity of Sal-B and Pue was evaluated in PC12 cells. The viability of the cells was higher than 90% when the concentration of Sal-B or Pue was below 400 μg·mL^−1^ (Figure S1). The protective effect of Sal-B, Pue and Sal-B+Pue in CoCl_2_ injured PC12 cells was studied. Before induced with CoCl_2_, PC12 cells were pretreated with Sal-B at series concentrations (1 μg·mL^−1^ to 100 μg·mL^−1^) and Pue (0.1 μg·mL^−1^ to 400 μg·mL^−1^) for 1 h, respectively. As indicated in Figure 2b–d, the viabilities of PC12 cells increased to 59.2 ± 5.78% (*p* < 0.01) at 70 μg·mL^−1^ for Sal-B and 50.5 ± 4.3% (*p* < 0.05) at 100 μg·mL^−1^ for Pue which were the highest in two groups, respectively. Then, 70 μg·mL^−1^ for Sal-B and 100 μg·mL^−1^ for Pue were chosen for the combination study. It is interesting to find that the cell viability was enhanced to 64.2 ± 8.14% after treated with Sal-B and Pue simultaneously with the ratio of 7:10 (Sal-B/Pue), which was significantly higher than the results treated with only one of them. The results demonstrated that the combination of Sal-B and Pue (7:10) had a better neuroprotective effect on neuron cell injury than Sal-B or Pue alone.

### 2.2. Combination of Sal-B and Pue Could Reduce ROS Level, Inhibit Apoptosis and Increase Mitochondrial Membrane Potential of CoCl_2_ Induced PC12 Cells

**Figure 3:**
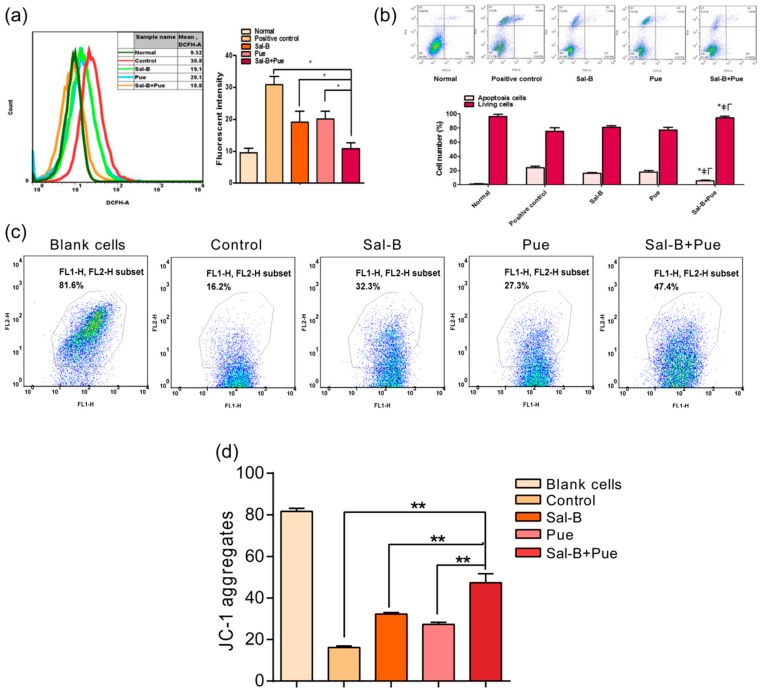
Effect of the combination of Sal-B and Pue on (**a**) ROS level; (**b**) apoptosis; (**c**) mitochondrial membrane potential on CoCl_2_ injured PC12 cells and (**d**) quantitative statistic result of JC-1 aggregates, representing the fluctuation of MMP. Data were expressed as mean ± SD (*n* = 3), * ≠Γ*p* < 0.05 Sal-B + Pue vs. positive control, Sal-B or Pue in apoptosis; * *p* < 0.05; ** *p* < 0.01 vs. other group.

As shown in Figure 3a, compared with the control group, the level of ROS could be significantly reduced by about 40% or 35% when treated with Sal-B (70 μg·mL^−1^) or Pue (100 μg·mL^−1^). The ROS level was further dropped by 65% when Sal-B (70 μg·mL^−1^) and Pue (100 μg·mL^−1^) were added simultaneously. The rate of living and apoptosis PC12 cells induced by CoCl_2_ were measured by flow cytometry assay. The rate of living cells was promoted from 64.3 ± 1.0% of the control group to 74.9 ± 0.6%, 75.1 ± 1.4% and 84.4 ± 0.9% (*p* < 0.05) of Sal-B group, Pue group and Sal-B + Pue group, respectively. The apoptosis rate of cells was 10.3 ± 0.8%, 4.0 ± 0.5%, 4.4 ± 1.2%, 3.9 ± 1.0% (*p* < 0.05) corresponding to control group, Sal-B group, Pue group and Sal-B + Pue group respectively (in Figure 3b). The rate of apoptosis was significantly reduced by more than sixty percent when the cells were pretreated with Sal-B + Pue before CoCl_2_ exposure. The protective effect of Sal-B + Pue group was also much stronger than that of Sal-B or Pue alone.

Mitochondria is one of the important energy production factories and also a major pathway of cerebral ischemia injury. When mitochondrial membrane potential (MMP) collapses, the fluorescence of JC-1 stained cells shifted from red (JC-1 aggregates) to green (JC-1 monomer), indicating the dysfunction of mitochondria. As seen in Figure 3c, the MMP of Sal-B + Pue group was much higher than that of Sal-B or Pue group. The quantitative results (Figure 3d) indicated that mitochondrial functions were effectively protected by the Sal-B and Pue and Sal-B + Pue achieved synergistic action.

### 2.3. Combination of Sal-B and Pue Decreased the Volume of Cerebral Infarction in Rats

**Figure 4:**
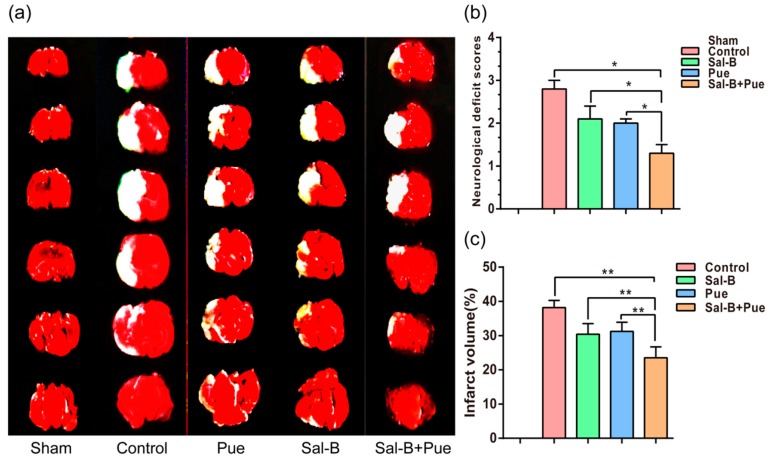
(**a**) Effects of Sal-B, Pue, Sal-B + Pue on infarct volume and neurological functional outcome (*n* = 6). (TTC staining) White area indicates infarcted brain tissue; (**b**) Neurological deficit score; (**c**) Percentage of cerebral infarction volumes were calculated as described in “Methods.” The Sal-B + Pue group exhibited obviously less cerebral infraction (white area) compared with other groups. * *p* < 0.05; ** *p* < 0.01 vs. other group.

To verify the effect of Sal-B, Pue and Sal-B + Pue in treating IS, middle cerebral artery occlusion (MCAO) model of rat was established. The drug solutions were administrated intravenously at 2 h post-reperfusion. The effect of the drugs on I/R rats was firstly evaluated by neurological deficit scores to all the experimental groups 2 h after reperfusion (Longa et al., 1989) [28]. The scores were assessed as following: 0: no motor disability (normal), 1: limbs weakness and cannot fully extend (mild); 2: circle to one side (moderate); 3: dump to one side (severe); 4: unconscious or unable to ambulate spontaneously (critical). As shown in Figure 4a, MCAO group induced the increase of score significantly in compare with sham group, indicating the model was built successfully. Both Sal-B and Pue could decrease the score effectively (*p* < 0.05). As expected, the combination of them could improve the therapeutic effect more than Sal-B or Pue alone (*p* < 0.05).

The TTC stained rat brain slices of different groups were shown in Figure 4b, we can see that the infarct volume was significantly reduced in all the three drug groups compared with the control. The infarct area was further calculated with Image J. The Figure 4c showed that if rats were performed without any treatment in the control group, the percentage of cerebral infarction volume was approximately 37.0 ± 2.3%. In the other group, Sal-B, Pue and Sal-B + Pue were injected 2 h after the reperfusion, the percentage of infarct volume was significantly reduced to approximately 31.6 ± 2.0%, 30.5 ± 1.3% and 24.4 ± 2.4% (*p* < 0.01). Among which, Sal-B + Pue had the strongest activity since the infarct volume in the group was much less than that in Sal-B group or Pue group (*p* < 0.01).

### 2.4. Combination of Sal-B and Pue Inhibited Neuronal Apoptosis and Relieved Injury of Ischemia Tissue in Rats

**Figure 5:**
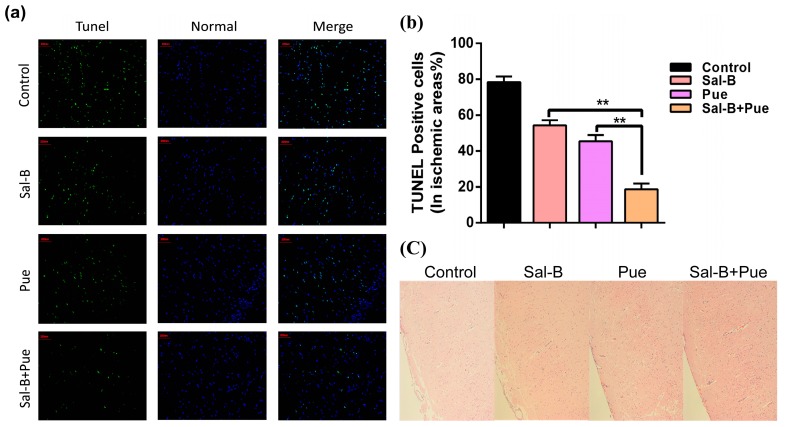
(**a**) TUNEL and (**c**) H&E microscopic images of brain sections 24 h after drug treatment. The green indicates the apoptotic cells and the blue represents normal cells in ischemic area (200 μm). The damaged neurons with apoptotic and necrotic changes were labeled (brown) at H&E staining and original magnification was 200×. (**b**) Quantitative results of apoptosis in ischemia area (*n* = 3), ** *p* < 0.01 vs. Sal-B or Pue.

To explore the necessity of the co-use of Sal-B and Pue, the neuroprotective effect of the drugs was further investigated by TUNEL and H&E staining to evaluate IS induced DNA fragmentation in neurons of ischemic region. TUNEL staining was used to evaluate apoptosis, in which the TUNEL cells were darkly stained and showed green fluorescence, representing the morphologic signs of apoptosis. As observed in Figure 5a, compared with other group, Sal-B + Pue group significantly reduced the green fluorescence. The apoptosis rate of Sal-B + Pue, Sal-B, Pue and PBS in control group was 18.7 ± 3.2%, 54.4 ± 2.8%, 45.5 ± 3.3% and 78.3 ± 3.1%, respectively (Figure 5b). The apoptosis of Sal-B + Pue group decreased significantly by approximately 4-fold compared with control group and more than 2-fold compared with Sal-B group or Pue group (*p* < 0.01).

The H&E staining (Figure 5c) was used for primary observation of histological changes of ischemic tissue in different group. In the control group, IS resulted in obvious neuronal damage and the neurons were arranged loosely. The nucleus boundary of some neurons was not clear and some neurons exhibited a shrinking appearance. The drug treatment alleviated the neuronal damage to different extent. Sal-B + Pue group showed the best effect.

### 2.5. Combination of Sal-B and Pue Attenuated Pro-Inflammatory Mediators mRNA and Protein Expression in Penumbra Region

**Figure 6:**
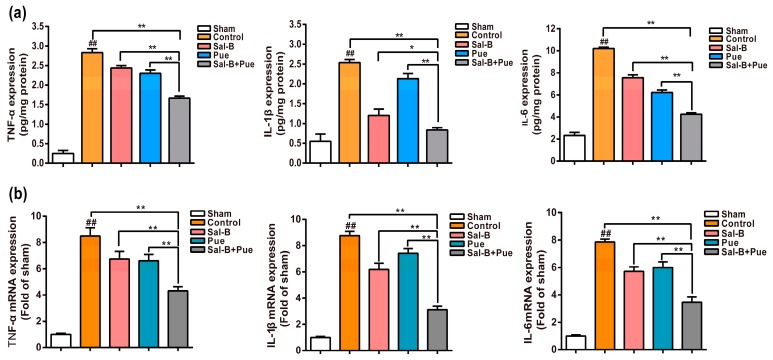
Effect of Sal-B, Pue and Sal-B + Pue treatment on (**a**) the protein expression and (**b**) mRNA levels of pro-inflammatory mediators TNF-α, IL-1β, IL-6 in the penumbra of the brain after 1.5 h MCAO and 24 h reperfusion injury (*n* = 6). The values were β-actin normalized and the relative mRNA levels in the sham-operated group were used as calibrators. Data are expressed as the mean ± SD (## *p* < 0.01 vs. sham; * *p* < 0.05 and ** *p* < 0.01 vs. control, Sal-B or Pue).

To evaluate the inhibitory effect of Sal-B + Pue on I/R-induced expression of pro-inflammatory molecules, the protein and mRNA expression levels of TNF-α, IL-1β, IL-6 in the penumbra were measured by ELISA and qPCR. As shown in Figure 6a, compared with the sham group, the levels of TNF-α, IL-1β, IL-6 in brain tissue were significantly increased in the control group (*p* < 0.01). However, compared to model group, both Sal-B and Pue could significantly decrease the levels of these mediators. The effect of Sal-B + Pue group was notably better than Sal-B and Pue (*p* < 0.01) for the three molecules.

The fluctuations in mRNA expression level of three pro-inflammatory genes were detected by quantitative real-time quantitative PCR. There were dramatic increases in gene mRNA expressions in the control group (Figure 6b) (*p* < 0.01), which were suppressed by the administration of Sal-B, Pue or the combination of them. Sal-B + Pue could specifically and significantly downregulate the level of TNF-α, IL-1β, IL-6 mRNA expression (*p* < 0.01). The effect was much stronger than that of Sal-B and Pue when used alone.

### 2.6. Combination of Sal-B and Pue Inhibited TLR4/MyD88 and SIRT1 Activation Signaling Pathway in Penumbra Tissue

**Figure 7:**
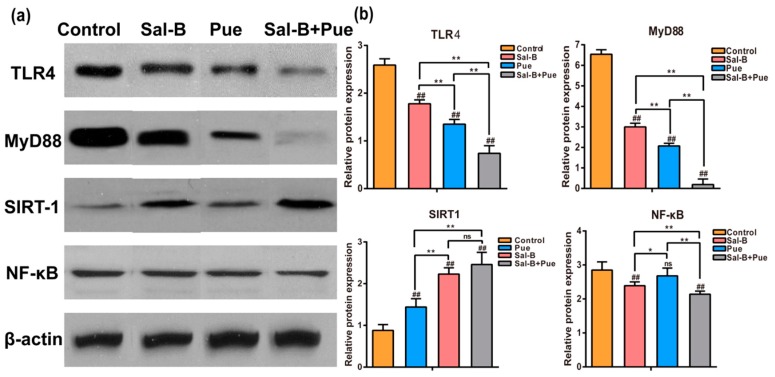
(**a**) Effects of Sal-B, Pue and Sal-B + Pue on the protein levels of TLR4, MyD88, SIRT1 and NF-κB in the brain tissue of penumbra (*n* = 3) after 1.5 h MCAO and 24 h reperfusion injury and (**b**) the statistical analysis results. Data are presented as mean ± SD. (## *p* < 0.01 each group vs. control group, “ns” represents non-significant difference; * *p* < 0.05; ** *p* < 0.01 vs. Sal-B or Pue).

As seen in Figure 7a,b, the expression of TLR4, MyD88, NF-κB in control (model) group was sharply higher than those in other groups (*p* < 0.01). The expression of SIRT1 in control group was markedly lower than those in other groups (*p* < 0.01). The protein expression levels of TLR4/MyD88 signaling pathway and the activation of SIRT1 could be significantly inhibited by both Sal-B and Pue. It is interesting to find that the inhibitory effect on TLR4/MyD88 protein expression of Pue was much stronger than that of Sal-B (*p* < 0.01). Meanwhile, the effect of Sal-B on SIRT1 activation was better than that of Pue (*p* < 0.01). The results elucidated that the two compounds might work on cerebral ischemia reperfusion injury through different ways. Therefore, the inhibitory effect was strengthened obviously by the combination of the two drugs, which could decrease neuroinflammation after ischemia by modulating innate and adaptive immunity through inhibiting the TLR4/MyD88 inflammatory signaling and SIRT1 activation pathways against brain injury.

## 3. Discussion

Traditional Chinese herbal formulas have been widely used for thousands of years to prevent and treat ischemic stroke based on their reliable therapeutic effect and low toxicity. However, the pharmacological mechanisms of the most herb compatibility are still unclear because of the complexity of herb components [29]. In the present study, two active components from the herb pair Danshen and Gegen were chosen since both of them are effective in treating ischemic stroke and often used together [30]. The optimum ratio of Sal-B and Pue was screened using base line proportional method. When the optimal concentration of Sal-B was confirmed to be 70 μg·mL^−1^, the combination of it with Pue of various concentrations (80~110 μg·mL^−1^) was designed and evaluated. Based on the results, the combination of 70 μg·mL^−1^ Sal-B and 100 μg·mL^−1^ Pue was proved of the highest effect and the ratio of 7:10 to be the optimum couple of them.

CoCl_2_ induced PC12 cell is a commonly used model for investigating cerebral ischemia reperfusion injury in vitro [31]. CoCl_2_ can induce the injury of neuronal cells and results in the generation of ROS, apoptosis and the transcriptional change of some genes such as hypoxia inducible factor (HIF-1α), p53, p21 and pDNA and therefore promotes the death of PC12 cells [32,33]. It was reported that CoCl_2_ could mimic the cerebral ischemia reperfusion injury conditions including the production of ROS in neuronal cells [34,35]. PC12 cells are similar in morphology, structure and function to neuron cells and have typical characteristics of sympathetic neuron cells. Therefore, we selected CoCl_2_ induced PC12 cells for cell viability study.

Our results in the present study showed that the administration of Sal-B (7 mg·kg^−1^, i.v.) and Pue (10 mg·kg^−1^, i.v.) at 2 h post-reperfusion could improve the neurological scores and decrease cerebral infarction volume through MCAO in rats. The combination of Sal-B and Pue attenuated ischemia-induced neuronal function dysfunction in vivo and reliably conferred a better protection against cerebral ischemia. The results were consistent with the previous reports of Sal-B or Pue alone [36].

During the pathophysiology process of IS, in addition to anoxia and energy failure, ion balance disorder, neuronal apoptosis, oxidative stresses, excitatory amino acid toxicity and inflammation occurs as well [37]. Briefly, the inflammation is considered to be a critical part of the cascade of events in the pathogenesis of stroke [38]. In the early stage of ischemic stroke, inflammation involves the activation of microglia and astrocytes, the influx of hematogenous cells recruited by adhesion molecules, the cytokines and chemokines across the activated blood vessel walls [39,40]. At the late stage, the expression of inflammatory cytokines, particularly TNF-α, IL-1β and IL-6, stimulates a complex cascade of events involving ICAM, P-selection, E-Selection, neutrophil activation, microglia/macrophage activation et al. [41]. What’s more, TNF-α, IL-1β and IL-6, are produced in neuron cells and often cause neuronal cells death via necrosis, apoptosis and autophagy [42,43]. Hence, inflammation is crucial in the process of ischemia stroke, as shown in Figure S2 (Supplementary data).

**Figure 8:**
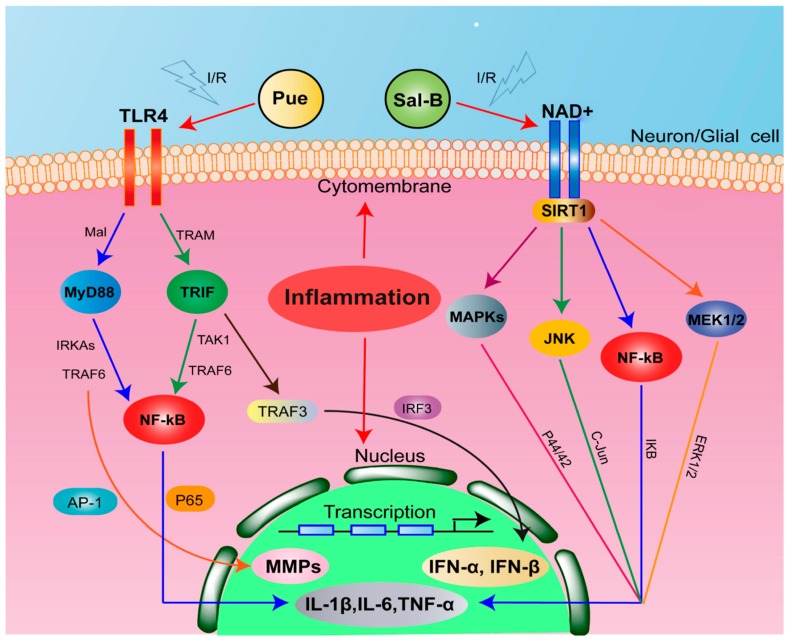
Schematic diagram for the neuroprotective effects of Sal-B + Pue in I/R injury. Sal-B + Pue suppressed TLR4/MyD88 signaling and SIRT1 activation to reach a synergistic effect after I/R injury.

TLR4, an innate and adaptive immune cell receptor of the pathogen recognition receptor family, plays a critical role in the progress of the neuroinflammation [44]. The increase of TLR4 protein expression in penumbra activates TRAF6 signaling with the MyD88 and subsequently further enhance the neuroinflammation response [45]. SIRT1, a member of the class III group of histone deacetylases, is thought to play an important role in neuroprotection against brain ischemia by deacetylation and inhibition of p53 and NF-κB-induced inflammatory pathways [46]. SIRT1 activation protects cells from oxidative stress-caused by cellular dysfunction [47]. In the present study, we find that both Sal-B and Pue exhibited potent activities to protect the brain from ischemia stroke. The combination of them could exert much stronger effect by reducing the inflammation and decreasing apoptosis in the penumbra. Molecular mechanism study demonstrated that Sal-B inhibited protein expression of TLR4 and MyD88, while Pue activated SIRT1 and downregulated expression of NF-κB, thus they reduced the gene expression of TNF-α, IL-1β, IL-6. The results suggested that Pue down-streamed obviously more TLR4 and MyD88 expression than Sal-B and Sal-B augmented SIRT1 expression and inhibited apoptosis and inflammation more than Pue. When used together, Sal-B and Pue could affect in different ways and realized a synergistic effect in the treatment of IS. Accordingly, the combination of Sal-B and Pue represents an effective and novel strategy for the prevention and treating IS in clinic. The possible mechanisms of their action could be illustrated as Figure 8.

## 4. Materials and Methods

### 4.1. Materials

Puerarin (purity > 98%), cobalt chloride (CoCl_2_), hydrogen peroxide and 2′,7′-dichlorofluorescin diacetate (DCFH-DA) were purchased from Dalian Meilun Biotech Co., Ltd. (Dalian, China). Salvianolic acid B (purity > 95%) was obtained from Shanghai Institute of Materia Medica (Shanghai, China), 5,5-Dimethyl-1-pyrroline N-oxide, 2,3,5-triphenyltetrazolium chloride (TTC), interleukin-1β (IL-1β), interleukin-6 (IL-6) and tumor necrosis factor (TNF-α) ELISA kits were provided by Neobio Company (Shanghai, China). Hematoxylin-eosin (H&E) staining kits were purchased from the Nanjing Jiancheng Institute of Biotechnology (Nanjing, China). Primer was obtained from Huajin Company (Shanghai, China). Rabbit anti-TLR4, rabbit anti-MyD88 rabbit anti-SIRT1 and rabbit anti-NF-κB were obtained from Proteintech, Inc. (Wuhan, China). 3-(4,5-dimethylthiazol-2-yl)-2,5-diphenyl tetrazolium bromide (MTT) were purchased from Sigma Aldrich (USA). Propidium iodide (PI) and annexin V-FITC were purchased from BioVision Inc. (Milpitas, CA, USA). PC12 cells were purchased from the Cell Bank at Chinese Academy of Sciences (Shanghai, China), Adult male Sprague-Dawley rats (250–300 g) were purchased from Sino-British BK Lab. Animal Co., Ltd. (Shanghai, China). The animal experiment protocol was approved by the Animal Experimentation Ethics Committee of Fudan University.

### 4.2. Cytotoxicity of Sal-B and Pue

In order to investigate the toxicity and find out the appropriate concentration of Sal-B and Pue to PC12 cells, the MTT assay was carried out firstly according to the manufacturer’s protocol.

PC12 cells (a rat pheochromocytoma cell line) were maintained in Dulbecco’s Modified Eagle’s Medium (DMEM) at 37 °C with 5% CO_2_. Sal-B and Pue were dissolved with DMSO and diluted to the final concentration with water. The final concentration of DMSO was less than 1%. PC12 cells were treated with Sal-B (10, 50, 100, 200, 400, 600 μg·mL^−1^) and Pue (10, 50, 100, 200, 400, 600 μg·mL^−1^) for 24 h, respectively, to evaluate the cytotoxicity of Sal-B and Pue. After incubation for 24 h, MTT (2 mg·mL^−1^) was added and incubated for 4 h. Then, the supernatant solution was removed and 150 μL of dimethyl sulfoxide (DMSO) was added in each well to form formazan dissolution. The optical density (OD, corresponding to absorbance) was measured with Microplate Reader at 570 nm. The cell viability was calculated as the following equation: cell viability (%) = ODexperimental group/ODcontrol group × 100%.

### 4.3. Effect of Sal-B and Pue against CoCl_2_ Induced Cell Injury

The injured cell model was established by adding CoCl_2_ to PC12 cells [35]. CoCl_2_ was dissolved in distilled H_2_O (10 mmol·L^−1^) and diluted to the final concentration with DMEM. PC12 cells at a density of 3 × 103/well were adhered to 96 well plates and then CoCl_2_ solution of different concentration (0.1, 0.2, 0.4, 0.6, 0.8, 1.0, 1.2, 1.5, 2.0 and 3.0 mmol·mL^−1^) was added and incubated for 24 h.

To test the protective effect of Sal-B and Pue against CoCl_2_ induced cell injury, PC12 cells were pretreated with various concentration of Sal-B (1, 50, 60, 70, 80, 90, 100 μg·mL^−1^) and Pue (0.1, 1, 10, 50, 100, 200, 400 μg·mL^−1^) for 1 h and then exposed to 1.2 mmol CoCl_2_ for 24 h. Then the cell viability was assayed with MTT.

### 4.4. Effect of Sal-B and Pue against ROS, Apoptosis and MMP In Vitro

PC12 cells were pretreated with Sal-B (70 μg·mL^−1^), Pue (100 μg·mL^−1^) alone, Sal-B and Pue (70 μg·mL^−1^ and 100 μg·mL^−1^) simultaneously for 1 h at a density of 3 × 105/well. Then, 1.2 mmol·L^−1^ CoCl_2_ was added and induced for 24 h. After that, the supernatant was replaced with serum free DMEM. DCFH-DA dilution (10 μM) was added into the cells, incubated for 30 min at 37 °C and resuspended with PBS (0.1 mmol) after washing twice with indicated inducers. Fluorescence intensity was detected by Flow Cytometry (FACS Calibur, BD, San Jose, CA, USA). 

Mitochondrial transmembrane potential (MMP, ΔΨm) was measured to evaluate mitochondrial functions. MMP was measured using a specific cyanine dye JC-1. The fluorescence of JC-1 stained cells shifted from red (JC-1 aggregates) to green (JC-1 monomer) while MMP breakdown. So, the JC-1 aggregates represented the fluctuation of MMP. JC-1 solution (5 μg·mL^−1^) was added into the cells at the second well, follow up in the same condition. 2′,7′-dichlorofluorescin diacetate dilution (10 μM) or Annexin-V (1 mg·mL^−1^) and PI (1 mg·mL^−1^) were added into cells at the third well as above. During Annexin-V and PI double staining assay, the fluorescence intensity was quantified with Flow Cytometry at 488 nm or 525 nm. 

### 4.5. Rat Model of Middle Cerebral Artery Occlusion (MCAO)

In order to explore the protective effect of Sal-B and Pue in vivo, MCAO model was established. The MACO model was performed according to literature [48]. Briefly, the rats (250–300 g) were anesthetized with 10% chloral hydrate (4 mL·kg^−1^) by intraperitoneal injection (i.p.) and tied to the operating table. The skin on the inside of the rat’s neck was cut along the line. Then the common carotid artery (CCA) was separated away from adjacent muscles and nerves to expose external carotid artery (ECA), CCA and ECA were ligated and internal carotid artery (ICA) was clamped to cut a small opening on one side with ophthalmic scissors. A monofilament nylon thread coated with poly-l-lysine was inserted into ICA via this small opening and advanced approximately 18–19 mm to the origin of the middle cerebral artery (MCA) at the near black spot. After that, the nylon thread was slowly withdrawn about 1mm while it encountered resistance and then the nylon thread was fixed [49]. After 1.5 h, the whole monofilament nylon thread was slowly withdrawn to allow reperfusion. Then, the skin was sewed and 1 mL of saline was administered via i.p. injection. 

### 4.6. Effect of Sal-B and Pue against IS In Vivo

The rats were randomly divided into following groups (*n* = 6): the sham, MCAO (as control), MCAO + Sal-B, MCAO + Pue, MCAO + (Sal-B + Pue). The sham operation group received the same surgery except for the insertion of nylon monofilaments. 1 mL PBS (0.1 mmol), instead of the drug solution, was given to the animals in MCAO control group. For the drug groups, Sal-B and Pue were dissolved in distilled water and injected (1 mL) from the tail vein. The doses of Pue, Sal-B and Sal-B + Pue were 10 mg·kg^−1^, 7 mg·kg^−1^ and 10 mg·kg^−1^ + 7 mg·kg^−1^, respectively. At 2 h post-reperfusion, the IS rats were administrated with Pue, Sal-B and Sal-B + Pue as descripted above. PBS (0.1 mmol) was added to the MCAO group. Approximately 24 h later, the rats were anesthetized with 10% chloral hydrate (i.p., 4 mL·kg^−1^) and their hearts were perfused with PBS for further study. Then, the brains were removed quickly and stored at −20 °C until analysis.

A portion of the IS rat’s brains were sliced into sections of 2 mm thickness. The sections were immediately stained with 1% triphenyltetrazolium chloride solution (TTC) at 37 °C for 20 min and fixed with 4% paraformaldehyde buffer for 10 min at room temperature. The infarction area was measured with Image J software and the infarction rate was calculated as white part area/whole brain red area × 100% [50], in which the white area and the red area represent the infarct area and the whole brain, respectively.

Neuronal apoptosis and brain tissue injury of the ischemia region were observed by TUNEL and hematoxylin-eosin (H&E) staining. Another part of the IS rat’s brains was slice into 2 μm thickness and fixed in 4% paraformaldehyde solution. Neuronal apoptosis was detected with TUNEL kit (Merck& Co. Inc., Kenilworth, NJ, USA) following the instruction. The percentage of apoptosis cells in ischemic area was calculated. The rate of apoptosis was calculated as: apoptosis cells/total cells in ischemic area × 100%. The cerebral ischemia tissues were stained with H&E solution according to standard procedures. The photographs (×200 magnification) were observed under optical microscope.

### 4.7. Effect of Sal-B and Pue on Inflammatory Cytokines In Vivo

The brains of all groups were taken out in −20 °C. The rat brain penumbra tissue (approximately 50 mg) were homogenized according to the instruction of ELISA kits (Neobioscience, Shanghai, China). The supernatant samples were obtained by centrifugation of the collected tissue (10,000 rpm for 10 min). The inflammatory cytokines, including TNF-α, IL-1β and IL-6, in the supernatant, were measured using commercially ELISA kits. 

### 4.8. Effect of Sal-B and Pue on mRNA Expression of TLR4, MyD88, NF-κB and TNF-α in Penumbra

Total RNA was isolated from the brain tissue of penumbra at the same position in the ipsilateral hemisphere using TRIzol reagent (Takara Bio, Shiga, Japan) in accordance with the manufacturer’s instructions. One microgram of total RNA was reverse transcripted using a one-step RT Kit (Takara Bio, Shiga, Japan) and the resulting cDNA was used as a PCR template for determining the mRNA expression level using an SYBR-green quantitative PCR kit (Takara Bio, Shiga, Japan) by real-time quantitative reverse transcription-PCR (RT-PCR). Comparative RT-PCR assays were performed for each sample in a final reaction volume of 20 μL, containing 10 μL SYBR-green fluorescent dye, 2 μL cDNA and 50 pmol each of the forward and reverse primers (Huagene, Shanghai, China). Rat specific primers for TNF-α, IL-6, IL-1β, β-actin were used (see supporting information Table S).

### 4.9. Effect of Sal-B and Pue on Protein Expression of TLR4, MyD88, NF-κB and TNF-α in Penumbra

Brain ischemia tissues were homogenized with a OMNI homogenizer (BioSpec Products, Inc., Bartlesville, OK, USA, level 10, 2 × 30 S) in lysis buffer (ingredients: 50 mmol·L^−1^ Tris-HCl buffer, pH 7.4, containing 0.1 mol·L^−1^ NaCl, 0.1% Triton X-100 and 1 tablet/10 mL complete miniethylenediamine tetraacetic acid–free protease inhibitor cocktail). The lysates were incubated for 30 min on ice and centrifuged at 10,000 rpm for 10 min. Supernatants were collected. The protein of this supernatant was measured using a BCA Protein Assay Kit (Beyotime, Shanghai, China). Proteins of 50 μg were separated on SDS-polyacrylamide gels and transferred onto a PVDF membrane (Millipore Corporation, Billerica, MA, USA). The membrane was blocked with 5% nonfat dry milk in Tris-buffered saline and then incubated with primary antibodies (TLR4, 19811-1-AP; MyD88, 66660-1-1g; SIRT1, 13161-1-AP and NF-κB/p65, 66535-1-1g5) (1:1000 dilution) overnight at 4 °C and horseradish peroxidase conjugated antibody at room temperature for 1 h. Protein expression was tested by western blot, with enhanced chemiluminescence (ECL) method and imaged using ChemiDoc XRS (BIO RAD, Hercules, CA, USA). Protein signals were quantified by scanning densitometry. The protein levels of pro-inflammatory mediators were expressed as relative integrated intensity normalized versus β-actin.

### 4.10. Statistical Analysis

The results were analyzed with Grafpad Software (GraphPad Software Inc., La Jolla, CA, USA). Statistical differences were evaluated using an unpaired Student’s *t*-test for the comparison of two groups and a one-way ANOVA for multiple-group comparisons. The data were expressed as mean ± standard deviation (X ± SD) and a *p* value < 0.05 was considered to indicate a significant difference.

## 5. Conclusions

In short, the study authenticated the rational compatibility of Sal-B and Pue in anti-inflammatory and neuroprotective effect against cerebral ischemia reperfusion insults by in vitro and in vivo studies. The possible mechanisms were associated with the inhibition of TLR4/MyD88 and SIRT1 activation signaling. The combination of Sal-B and Pue provides a potent and promising strategy for the treatment of ischemic stroke and is worthy for further development.

## Supplementary Materials

Supplementary materials are available online. Figure S1: Effects of Sal-B (10, 50, 100, 200, 400, 600 μg·mL^–1^) and Pue (10, 50, 100, 200, 400, 600 μg mL^–1^) on the survival rate of PC12 cells by MTT assay (*n* = 6). Each Value represents the mean ± SD, Figure S2: The status of inflammation in pathological process of ischemia stroke. Table S: The primer sequences used for RT-PCR assay.
