# Supplementary material for: Synergistic Effects of Salvianolic Acid B and Puerarin on Cerebral Ischemia Reperfusion Injury
Source: Molecules. 2018 Mar 2;23(3):564. doi: 10.3390/molecules23030564 (PMC6017479; doi:10.3390/molecules23030564)
Supplement: Supplementary file 1 [file molecules-23-00564-s001.pdf]

Synergistic effects of Salvianolic acid B and Puerarin on cerebral ischemia reperfusion injury

Chengli Ling[†#], Jianming Liang[#], Chun Zhang[#], Ruixiang Li[#], Qianqian Mou[†],

Jin Qin[#], Xiaofang Li[†*], Jianxin Wang[#*]

Table S. The primer sequences used for RT-PCR assay.

Gene	Species	Forward Primer	Reverse Primer
TNF-α	Rat	TACTCCCAGGTTCTCTTCAAGG	GGAGGCTGACTTTCTCCTGGTA
IL-1β	Rat	CACCTCTCAAGCAGAGCACAG	GGGTTCCATGGTGAAGTCAAC
IL-6	Rat	GAGTTGTGCAATGGCAATTC	ACTCCAGAAGACCAGAGCAG
β-actin	Rat	ATCGTGGGCCGCCCTAGGCACC	CTCTTTAATGTCACGCACGATTTC

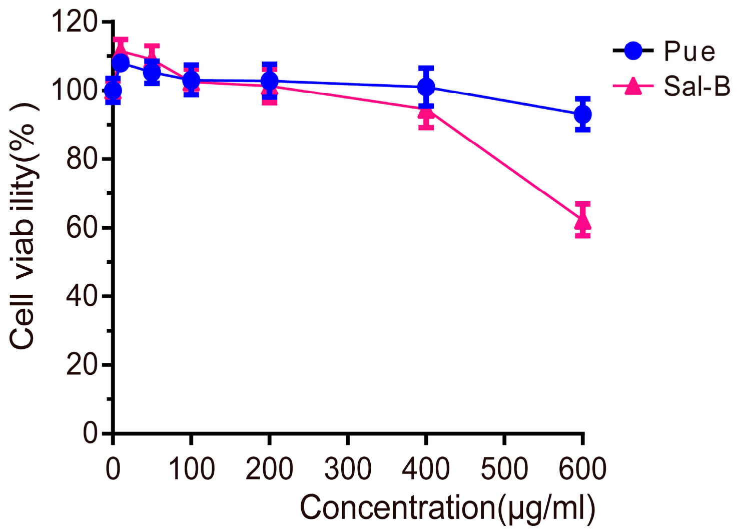

Figure S1. Effects of Sal-B (10, 50, 100, 200, 400, 600 μg·ml^{-1}) and Pue (10, 50, 100, 200, 400, 600 μg·ml^{-1}) on the survival rate of PC_{12} cells by MTT assay (n=6). Each Value represents the mean ± SD.

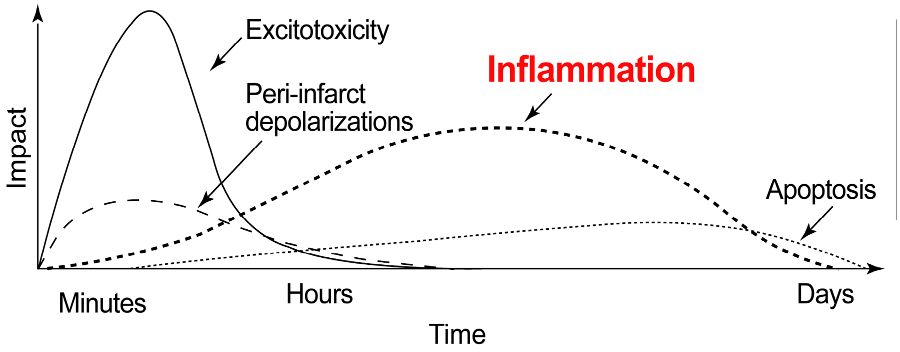

Figure S2. The status of inflammation in pathological process of ischemia stroke.
